# Thin-Film Solar Cells Based on Selenized CuSbS_2_ Absorber

**DOI:** 10.3390/nano11113005

**Published:** 2021-11-09

**Authors:** Minghao Zhao, Junsheng Yu, Lijuan Fu, Youwei Guan, Hua Tang, Lu Li, Jiang Cheng

**Affiliations:** 1 School of Optoelectronic Science and Engineering, University of Electronic Science and Technology of China (UESTC), Chengdu 610054, China; swamphao@163.com (M.Z.); jsyu@uestc.edu.cn (J.Y.); juan956545@hotmail.com (L.F.); lli@cqwu.edu.cn (L.L.); 2 Co-Innovation Center for Micro/Nano Optoelectronic Materials and Devices, Chongqing University of Arts and Sciences, Chongqing 402160, China; htang44@163.com

**Keywords:** thin film solar cells, CuSbS_2_, CuSbS_2_(Se), spray pyrolysis deposition, selenylation

## Abstract

Copper antimony sulfide (CuSbS_2_) has attracted significant interest as an earth-abundant photovoltaic absorber. However, the efficiency of the current CuSbS_2_ photovoltaic device is too low to meet the requirement of a large-scale application. In this study, selenylation was introduced to optimize the band structure and improve the device performance. Selenized CuSbS_2_ [CuSbS_2_(Se)] films were realized using porous CuSbS_2_ films prepared by spray deposition with a post-treatment in Se vapor. The as-prepared CuSbS_2_(Se) films exhibited a compact structure. X-ray diffraction and elemental analysis confirmed the effective doping of Se into the lattice by substituting a part of S in CuSbS_2_. Elemental analysis revealed a gradient distribution for Se from the top surface to the deeper regions, and the substitution rate was very high (>39%). Dark J–V characteristics and AC impedance spectroscopy analysis showed that selenylation significantly reduced the carrier recombination center. As a result, the selenized CuSbS_2_ device exhibited a significant efficiency improvement from 0.12% to 0.90%, which is much higher than that of the simply annealed device (0.46%), indicating this technique is a promising approach to improve the performance of CuSbS_2_ solar cells.

## 1. Introduction

To cope up with the challenges brought by increasing global energy consumption and pollution caused by fossil fuel consumption, the utilization of solar energy such as the photovoltaic technique has received extensive research attention for a long time [1,2]. In the last decade, inorganic thin-film solar cells have achieved a great height via material innovation and technical improvement. Cadmium telluride (CdTe) and copper–indium–gallium–selenium (CIGS) thin-film solar cells have achieved tremendously high efficiency of >21% [3,4]. The rapid transcendence of organic–inorganic metal halide perovskite solar cells to above 25% efficiency mark has captivated the significant attention of the photovoltaic research community [5,6]. Very recently, Hou’s group reported the preparation of organic solar cells with a certified 19.6% high efficiency via the tandem technique, representing that the breakthrough of 20% efficiency for organic solar cells is simply a matter of time [7]. Nevertheless, this achievement does not indicate that most problems of larger-scale application have been well solved. Instead, many scientists still thrive on overcoming the obstacles of stability and lifetime of existing high-efficiency devices or seeking new high efficiency, environmentally friendly, and chemically stable photovoltaic materials.

Antimony chalcogenides, including Sb_2_Se_3_, Sb_2_S_3_, and Sb_2_(S_1−*x*_Se*_x_*)_3_, have attracted significant research attention as a new group of absorber materials that meet the most requirements for large-scale application, in particular, the chemical stability and raw materials source aspects [8,9,10,11,12,13,14]. Unencapsulated devices based on antimony chalcogenides remain nearly undegraded after two months in ambient air. They can even work fine in damp–heat conditions (85 °C, 85% humidity). For example, the Sb_2_(S_1−*x*_Se*_x_*)_3_ device prepared by spray pyrolysis exhibited an efficiency degradation of only less than 6% in that condition. Through years of continuous efforts and development, the best power conversion efficiency (PCE) of antimony chalcogenides-based devices has exceeded 10%, indicating it has great potential as an alternative next-generation solar cell material.

Copper antimony chalcogenides, such as CuSbS_2_, CuSbSe_2_, and CuSb(S,Se)_2_ have also been considered as the promising photovoltaic absorbers due to their tunable energy bandgap of 1.45–1.08 eV, switchable hole concentrations of 10^16^–10^18^ cm^−3^, and hole mobility of 0.1–1.0 cm^2^ V^−1^ S^−1^ [15]. Copper antimony chalcogenides consist of a cleavage-free chalcostibite structure. The devices exhibit very high stability, which is even better than that of antimony chalcogenides. Comparative analysis indicates that antimony chalcogenides have a layered stibnite structure in which the ribbons stacked perpendicular to [001] direction are held together by the weak van der Waals forces, which could undoubtedly bring unstable factors [16,17]. Unfortunately, most copper antimony chalcogenide solar cells have suffered from poor (<4%) efficiencies, which is far from its spectroscopic-limited maximum efficiency (22.9%) [1,18]. However, some limiting factors for the unexpected low efficiencies have become progressively clearer after years of research efforts. The interface problems such as ohmic contacts [19] and band offset [20,21] between the CuSbS_2_ and CdS buffer layer have been excessively discussed. In our previous study, it was found that crystal defects and atomic disorders could also bring serious recombination [22]. Although the PCE of the CuSbS_2_ device was improved from 0.73% to 2.48% by introducing Ag atoms into CuSbS_2_ crystal structure, there still existed many unsettled problems. For example, the energy band (E_g_) of CuSbS_2_ of 1.58 eV is a little larger for an efficient solar cell. The current density (*J_SC_*) was restricted to a very low level due to the narrow absorption range (350–600 nm) and the poor carrier transport capacity was encountered. 

Herein, in order to improve the performance of CuSbS_2_ devices, selenylation treatment was taken into account. High-quality CuSbS_2_(Se) thin films were successfully realized by the previously used spray pyrolysis-selenylation (SPS) method. This routine was selected herein because spray-coated CuSbS_2_ films may possess a better porosity that could make selenylation easier and create a higher-quality CuSbS_2_(Se) film than that prepared by other methods. As expected, the selenized CuSbS_2_ absorbers exhibited obvious optimized optical and electrical properties, bringing a significant improvement of PCE from 0.12% to 0.90%, which is obviously higher than that treated in N_2_ at the same temperature. Although the progress is not significant, this method has been proven to be very effective in improving CuSbS_2_ solar cells.

## 2. Materials and Methods

### 2.1. Chemicals

Copper acetate monohydrate (C_4_H_6_CuO_4_·H_2_O, 99.95%), antimony acetate (C_6_H_9_O_6_Sb, 99.99%), thiourea (CH_4_N_2_S, 99%), glycol methyl ether (C_3_H_8_O_2_, 99.5%), and selenium powder were purchased from Macklin Inc. (Shanghai, China).

### 2.2. Preparation of CuSbS_2_(Se) Method

CuSbS_2_(Se) was prepared by the previously reported SPS method, i.e., CuSbS_2_ was first prepared by spray pyrolysis deposition (SPD) and then selenized in Se vapor. For the SPD of CuSbS_2_, the spray solution was prepared by dissolving copper acetate monohydrate (0.120 g, 0.6 mmol), antimony acetate (0.179 g, 0.6 mmol) and thiourea (0.190 g, 2.5 mmol) in glycol methyl ether (10 mL). To prevent precipitation, copper acetate monohydrate and antimony acetate should be respectively dissolved in glycol methyl ether at first. Further several drops of acetic acid (0.3 mL) should be added to the solution to inhibit hydrolysis.

In CuSbS_2_ deposition processing, the spray solution atomization rate was accurately tuned at 0.3 mL min^−1^, while the carrier gas (N_2_) was introduced at a flow rate of 20 L min^−1^, and the substrate temperature was maintained at 350 °C. The deposition rate of CuSbS_2_ films was about 100 nm min^−1^. Then, the prepared CuSbS_2_ films were transferred to a controlled dual-temperature zone RPT furnace. The selenylation process was fine-tuned to a condition where Se powder was maintained at 350 °C and the temperature of CuSbS_2_-coated wafer was maintained at 400 °C. 

### 2.3. Device Fabrication

Indium tin oxide (ITO) coated glasses with a nominal sheet resistance of 7 Ω were used as substrates for coating fabrication. All the substrates were cleaned with alkaline detergent (RM10-07, Rigorous Co., Ltd., Shenzhen, China) in an ultrasonic bath prior to their use. A cadmium sulfide (CdS, 100 nm) thin film was deposited on ITO by the widely used chemical bath deposition (CBD) technique. Then, a 1200 nm thick CuSbS_2_ thin film was deposited on the CdS buffer layer by SPD. Next, the CuSbS_2_ thin film was selenized in the RPT furnace for several minutes. Finally, 60 nm-thick Au back-contact electrodes were sputtered onto the top of CuSbS_2_(Se) in a DC sputtering coater.

### 2.4. Films Characterization and Device Testing

The composition and crystal structure were characterized by X-ray diffraction (XRD, AXS D8 Advance, Bruker, Karlsruhe, Germany). The film morphologies were characterized by scanning electron microscopy (SEM, Merlin, Zeiss, Oberkochen, Germany). Optical transmittance spectra of the blend films were obtained using an ultraviolet–visible–infrared (UV–vis–IR) spectrophotometer (Cary 5000, Agilent, Santa Clara, CA, USA). The electronic structure and energy level films were analyzed by X-ray photoelectron spectroscopy and UV photoelectron spectroscopy (XPS and UPS, respectively, ESCALAB 250xi, ThermoFisher, Waltham, MA, USA). The thickness of the films was measured using a stylus profile meter (Alpha-Step D-100, KLA-Tencor, Milpitas, CA, USA).

The current density–voltage (J–V) characteristics were measured using a Keithley 2400 SourceMeter, under the illumination of a xenon lamp (94043A, Newport, Rhode Island, USA) with a power of 100 mW cm^−2^. External quantum efficiency (EQE) was measured using an integrated system (7-SCSpecIII, Beijing SOFN, Beijing, China) with a lock-in amplifier under short-circuit conditions. Carrier transporting behavior was investigated by electrochemical workstation (CHI760e, Chenhua, Shanghai, China) under a suitable open-circuit voltage.

## 3. Results and Discussions

**Figure 1:**
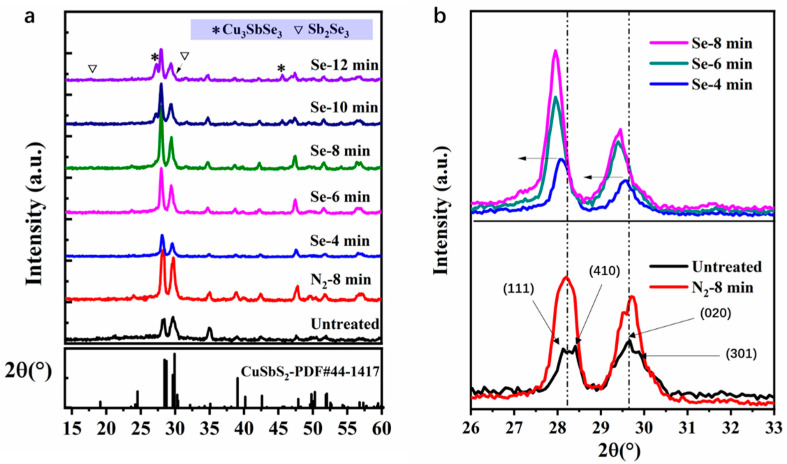
Crystal structure and composition analysis: (**a**) XRD analysis of the untreated film, and films annealed in N_2_ and Se vapor and (**b**) Magnified XRD peaks of the annealed film and films selenized for 4, 6, and 8 min.

The formation of CuSbS_2_(Se) thin films was largely dependent on the quality of CuSbS_2_ thin films and the selenylation process. Moreover, the selenylation should bring multidimensional effects on the absorber and devices. Before the device analysis, the crystal structure and morphologies of selenized CuSbS_2_ were first investigated. The composition and phase information of CuSbS_2_(Se) selenized for different durations were confirmed by XRD. Figure 1a shows the XRD patterns of seven films subjected to different treatments. The XRD pattern of the untreated sample, in general, matched that of chalcostibite CuSbS_2_ (JCPDS No. 44-1417). The amorphous-like XRD peaks reflect its poor crystallinity. The sample heated in N_2_ at 400 °C for 8 min shows a higher intensity, indicating that a post-annealing for SPD prepared CuSbS_2_ is necessary. The samples selenized at 400 °C for a short time (~8 min) show a spectrum similar to that treated in N_2_. However, when the selenylation duration was prolonged to 10 and 12 min, Cu_3_SbSe_3_ and Sb_2_Se_3_ impurities peaks could be clearly observed. Similar to the other selenized absorbers, CuSbS_2_(Se) is unstable in Se-rich conditions and the selenylation duration should be controlled at a reasonable range [23,24]. Figure 1b shows the magnified view of the XRD peaks at 26–33°. The peaks centered at low 2θ correspond to the combination of (111) and (410) planes and the other peaks centered at higher 2θ indicate a combination of (020) and (301) planes. The two peaks exhibit a significant enhancement in intensity, but no obvious shift is observed before and after treatment in N_2_. For the selenized samples, all the four peaks gradually shift to lower 2θ with increasing full width at half maximum (FWHM) with the increase in the selenylation duration from 4 to 8 min, indicating the successful accessibility of Se to the crystal lattice.

**Figure 2:**
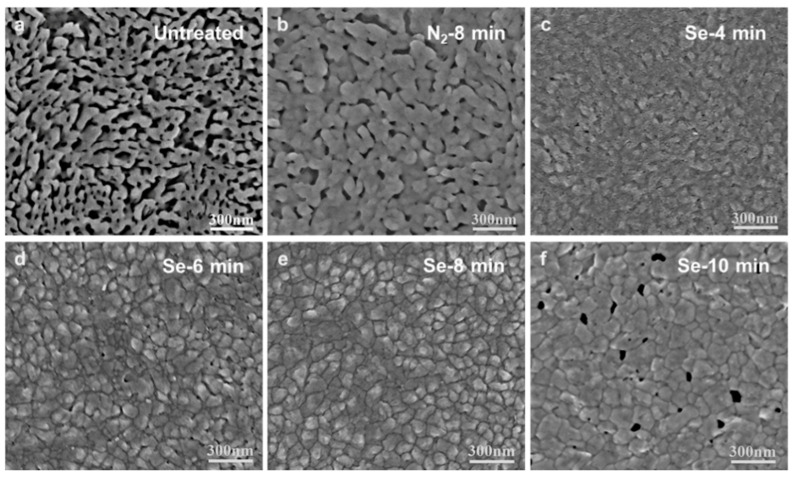
Analysis of surface morphologies of CuSbS_2_ film: SEM micrographs of (**a**) the untreated film, (**b**) film annealed in N_2_ for 8 min, and selenized films for (**c**) 4 min, (**d**) 6 min, (**e**) 8 min, and (**f**) 10 min.

Surface morphologies further revealed the variation of chemical and crystal structure that was affected by post-treatment. Figure 2 shows the top SEM image of untreated CuSbS_2_ thin films and CuSbS_2_ treated in N_2_ and Se vapor for varied duration. The untreated films (Figure 2a) exhibit a porous and incompact structure. This loose texture provided a large reaction contact area. In contrast, the selenylation reaction level could be largely restricted by the compact structure of film because it was not so easy for the Se element to transport from the surface to the deeper regions. For example, selenized Sb_2_S_3_ devices that are prepared by thermal evaporation always exhibit much lower PCE than those obtained by solution method because of their compact surface and big crystal grains [11,23,25]. Figure 2b shows the surface of the sample treated in N_2_ for 8 min. Both the size and density of pores decreased, and grains were found to be bonded to each other in all ranges. With 4–6 min selenylation time, no big pores could be found anywhere. The crystal grains grew bigger and the boundary gradually became more and more distinct when the selenylation duration prolonged from 4 to 8 min. It also indicates that the crystallinity of CuSbS_2_ thin films could be improved by the heat treatment at 400 °C [26,27], which agrees well with the XRD results. The films selenized for 8 min showed a smooth and compact surface with an average grain size of about 70 nm. However, when increasing the selenylation duration (>12 min), pinholes could be found everywhere, which could lead to serious short circuits in devices.

**Figure 3:**
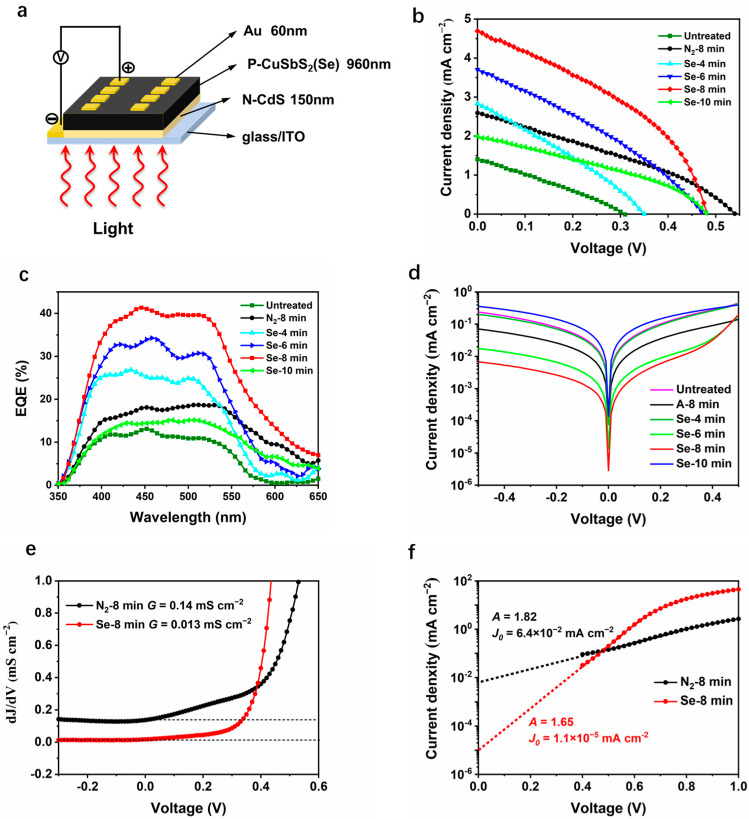
Performance test of the devices based on CuSbS_2_ and CuSbS_2_(Se): (**a**) the structure of CuSbS_2_(Se) solar cells; (**b**) J–V characteristics under illumination, (**c**) EQE, (**d**) J–V characteristics in dark of CuSbS_2_ device and CuSbS_2_(Se) devices selenized for different durations; (**e**) shunt conductance G characterizations, (**f**) ideality factor A and reverse saturation current density *J*_0_ characterizations.

To evaluate the performance of CuSbS_2_(Se) thin films as absorbers, solar cells with a simple structure of ITO/CdS/CuSbS_2_(Se)/Au were prepared, and its sketch is illustrated in Figure 3a. Each specimen consists of eight devices with an active area of 0.04 cm^2^. All the specimens were tested under the same conditions. The current density–voltage (J–V) characteristics and external quantum efficiency (EQE) curves of the devices prepared by different treatments are shown in Figure 3b,c, and their performance details are presented in Table 1. The devices with untreated CuSbS_2_ absorber exhibit a *V_OC_* of 0.31 V, a *J_SC_* of 1.41 mA cm^−2^, and a fill factor (FF) of 0.28, resulting in a very low average PCE of 0.12%. The devices with absorbers heated in N_2_ exhibit better performance than the untreated devices due to the improvement of crystallinity. The PCE increases to 0.46 ± 0.05% with a much higher *V_OC_* of 0.54 V and a *J_SC_* of 2.6 mA cm^−2^. Overall, all the devices using selenized absorbers show varying degrees of enhancement in photovoltaic performance. With 4-min slight selenylation, the *J_SC_* and *V_OC_* increased to 2.83 mA cm^−2^ and 0.35 V, respectively. The device with optimum performance was obtained when the absorbers were selenized for 8 min. The highest PCE of 1.04% was achieved with a high *J_SC_* of 5.31 mA cm^−2^. When the selenylation duration was prolonged to 10 min, PCE further reduced to 0.31 ± 0.17% with a much lower *J_SC_* and a poor yield. When the selenylation duration was lengthened to 12 min or longer, few available devices were detected from J–V characteristics curve. This degradation could be caused by pinholes or even cracks as shown in Figure 2f. The same situation existed in selenized Sb_2_S_3_ and AgSbS_2_ devices [23,24]. The morphology damage could be related to Ostwald ripening, as the distribution of the Se element occurs faster than that of the S element. Thus, the selenylation temperature and duration for CuSbS_2_ film should be accurately controlled (e.g., 6–8 min in this condition).

EQE can provide more information about the photo-response for devices using CuSbS_2_ that are treated under different atmospheres and for different durations [28]. The devices with untreated CuSbS_2_ absorber exhibit a low EQE (>10%) with a narrow photoresponse range of 350–600 nm. With the increase in the selenylation duration from 4 to 8 min, the devices show increasing EQE from 15% to 40%, which agrees well with the *J*_SC_ trend. The edge of the photo-response range shows a slight increase from 600 nm to more than 650 nm. Noteworthy, the EQE of CuSbS_2_ devices treated in N_2_ is obviously broader than those of untreated CuSbS_2_ devices and devices selenized for a short time (<6 min), although its EQE is even lower than the devices selenized for 4 min. It indicates that the low EQE at long wavelength range (600–650 nm) for devices using untreated CuSbS_2_ film and CuSbS_2_(Se) films selenized for a short time, is caused by interfacial carrier combining probably with the low crystallinity because the energetic photons are largely absorbed near the CdS/CuSbS_2_ interface [26].

To further reveal the mechanisms of selenized devices, dark J–V characteristics were investigated for devices subjected to different treatments. Figure 3d demonstrates that the devices selenized for 8 min show the lowest leakage current, illustrating its low charge carrier recombination. Through closer observation, the device treated in N_2_ exhibits a higher leakage current, although they have a similar crystallinity as selenized devices. To further explore the device physics, shunt conductance (*G*), series resistance (*R*), diode ideality factor (*A*), and reverse saturation current density (*J*_0_) of devices treated in N_2_ and Se vapor were calculated according to the general single exponential diode equations [29] presented below:

(1) $$J=J_{0}exp\left[\frac{q}{AKT}\left(V-RJ\right)\right]+GV-J_{L},$$

(2) $$\frac{dJ}{dV}=J_{0}\frac{q-Rs\frac{dJ}{dV}}{AkT}exp\left[\frac{q\left(V-JRs\right)}{AkT}\right]+G,$$

where *J_L_* is photocurrent density, *k* is Boltzmann constant, *T* is temperature, *R_s_* is the series resistance, *R_sh_* is the shunt resistance, and *G* = 1/*R_sh_* is the shunt conductance. The direct plot of *dJ*/*dV* against *V* is presented in Figure 3e, where the *G* values obtained were 0.14 and 0.013 mS cm^−2^ for devices treated in N_2_ and Se vapor, respectively, by reading from the flat regions under reverse bias. The *J*_0_ values could be determined by the extended line of the linear region at 0 V, and the A values were obtained by the slopes of the lines. Figure 3f exhibits that the selenized devices have a very low *J*_0_ value of 1.1 × 10^−5^ mA cm^−2^ compared to the devices heated in N_2_ (6.4 × 10^−2^ mA cm^−2^), confirming its significant low carrier recombination. The *A* value can reflect the main recombination region in the p–n junction. For an ideal p–n junction, *A* value is 1. Both the devices treated in N_2_ and Se vapor show *A* values between 1 and 2, indicating the main recombination occurs at the space charge region. For a selenized device, a smaller *A* value (1.65) represents that the absorber has a much lower trap density. Compared with devices heated in N_2_, the electrical improvement herein should be attributed to the variation of composition and crystal structure, accompanied by an essential improvement in the carrier concentration and carrier transport efficiency for the films.

**Figure 4:**
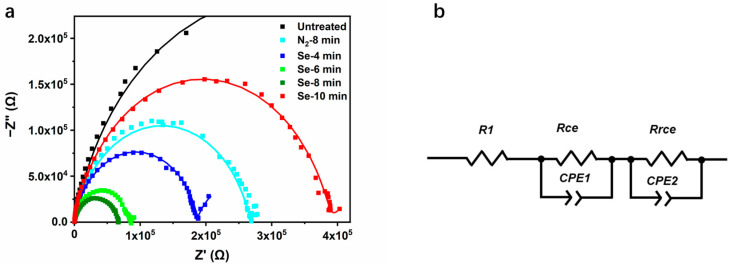
ESI analysis: (**a**) Cole–Cole plots and (**b**) equivalent circuit model of CuSbS_2_ and CuSbS_2_ (Se) devices. The solid lines represent the fitted results.

To further understand the effect of selenylation on the carrier dynamics, EIS measurements were carried out, and the results (Cole–Cole plots) in the frequency range 10 Hz to 1 MHz are shown in Figure 4a [30,31]. The plots of all devices exhibited a quasi-semicircle shape which could be divided by a high-frequency arc and a connected low-frequency arc. The Cole–Cole plots were analyzed using an equivalent circuit as shown in Figure 4b in which *R1* represents the series resistance, shunt pair *Rce* and *CPE1* are associated with CuSbS_2_(Se)/CdS interfaces, and shunt pair *Rrce* and *CPE2* represents the carrier transport factor in CuSbS_2_(Se). *CPE* represents a capacitance-like element in the modeling for inhomogeneity in the interface, and is conventionally defined by two values, namely, *CPE-T* and *CPE-P*. *CPE-T* is capacitance, while *CPE-P* is a non-homogeneity constant. The results indicate that the plots are in reasonable concordance with the simulated curve (the solid line). All the parameters calculated from the fitted curve are summarized in Table 2. 

It is well known that the series resistance is a factor that largely affects the photocurrent of a solar cell. For the untreated devices, the value of *R1* is 265.7 Ω, and it significantly decreases to 58.7 Ω for the devices selenized for 8 min, corresponding to a substantial improvement of *J_SC_*, increasing from 1.41 to 4.70 mA cm^−2^. The devices selenized for 8 min also exhibit the lowest *Rce*, indicating their lower interfacial resistance. In contrast, the values of *R1* and *Rce* remain as 212.5 Ω and 2.66 × 10^5^ Ω, respectively, and the *J_SC_* of devices show a very limited increase to 2.60 mA cm^−2^. *Rrce,* a characteristic parameter, reflects the recombination resistance [32]. A large value of *Rrce* represents that the device has a low carrier recombination rate. Notably, the *Rrce* of untreated devices is 0.8 × 10^5^ Ω and it increases to 1.2 × 10^5^ Ω and 8.1 × 10^5^ Ω after heated in N_2_ and Se vapor for 8 min, respectively. It indicates that the simple heat treatment has little effect on the carrier transport, and selenylation plays a more important role in reducing the carrier recombination. As previously discussed, the deep level crystal defects S vacancies (V_S_) constitute the dominant recombination centers that have profound effects on the electronic structure and carrier mobility of CuSbS_2_ film [29]. The density of V_S_ obviously reduces as some Se gets into crystal lattice when it is selenized for a short time. However, when the selenylation duration is too long (≥10 min), the recombination rate increases sharply due to the short-circuit caused by the pinholes. The total carrier recombination rate for the devices could be reflected by a voltage-dependent time constant (*τ_avg_*), which can be defined by the following equation [33]:

(3) $$\tau_{avg1}=CPE1-T\times R1$$

Table 2 summarizes that the *τ_avg_*_1_ that is related to interface for 8 min-selenized devices is 0.60 ms, which is a little higher than that of the devices treated in N_2_. However, *τ_avg_*_2_ that is related to carrier transport in CuSbS_2_ for 8 min-selenized devices is 33.88 s, which is much higher than that of the devices treated in N_2_ (3.80 s), indicating selenylation has a greater effect on the electronic properties of CuSbS_2_ film than CuSbS_2_(Se)/CdS interfaces.

**Figure 5:**
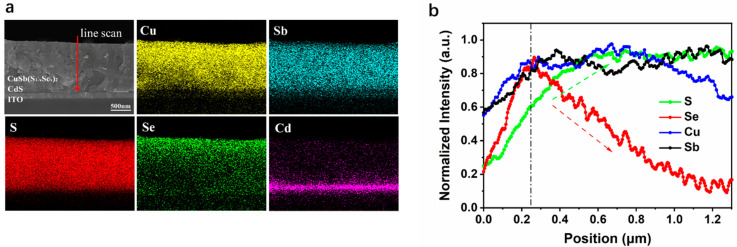
Quantitative elemental analysis: (**a**) EDS elemental mapping and (**b**) Element distribution in CuSbS_2_(Se) selenized for 8 min.

**Figure 6:**
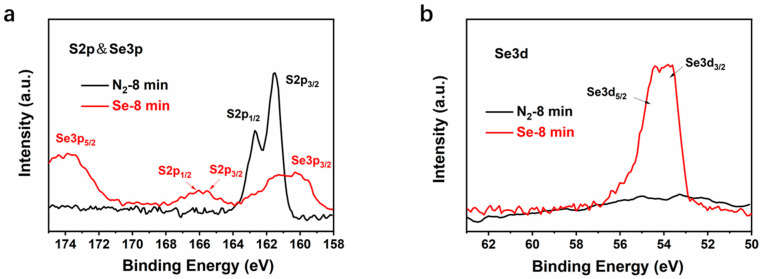
XPS analysis: Core level spectra of (**a**) S2p and Se3p and (**b**) Se3d of CuSbS_2_ and CuSbS_2_(Se) selenized for 8 min.

To further study the mechanism of selenylation and reveal the characteristics of reaction between Se and CuSbS_2_ films, quantitative elemental analysis was carried by X-ray energy dispersive spectroscopy (EDS). The global concentration of each element on CuSbS_2_(Se) films selenized for different durations was also analyzed. Table 3 summarizes the concentration and ratio of Cu, Sb, S, and Se, and the data corresponding to each element are the average of 6 test points. The results indicate that a small amount of Se (<1%) could be found in 4-min selenized film. The Se concentration increased gradually with the increase in the selenylation time to 10 min. When the selenylation duration was prolonged to 6 and 8 min, a lot of S element in CuSbS_2_ was substituted by Se. Moreover, the compositional ratio Se/(S + Se) reached 24% and 39%, confirming that CuSbS_2_ prepared by SPD method could easily react with Se vapor and transformed to CuSb(S,Se)_2_ at 400 °C. A cross-section view along with EDS elemental mapping results of 8-min selenized CuSbS_2_(Se) film is shown in Figure 5a. Figure 5b exhibits the line scanning results of S, Se, Cu, and Sb elements. The concentration of Se element decreases gradually from surface to deeper inside the film, while the concentration of S at near-surface is obviously lower than that in the inner body. It indicates that the most of S element was substituted with Se in the near surface of CuSbS_2_(Se) film, and the substituted rate reduced gradually with the increase in the diffusion length. The XPS spectra of S and Se provide another evidence, as shown in Figure 6a,b. For 8-min annealed CuSbS_2_ film, a sharp S2p XPS peak centered at 162 eV consisted of S2p_1/2_ and S2p_3/2_ could be well distinct. However, this peak became very weak even lower than those of Se3p_1/2_ and Se3p_3/2_ with an 8-min selenylation. Instead, the sharp Se3d XPS centered at 54.2 eV and consisting of S3p_3/2_ and S3p_5/2_, was easily found in 8-min selenized films, confirming the existence of few S elements in the near surface region. Significantly, the concentration of Se near the interface is less than 10%, which could explain why there is not much difference in photo-response range (Figure 3c) and why selenylation has a greater effect on the electronic properties of CuSbS_2_ film than CuSbS_2_(Se)/CdS interfaces (Se AC impedance analysis) for devices treated in N_2_ and Se vapor.

**Figure 7:**
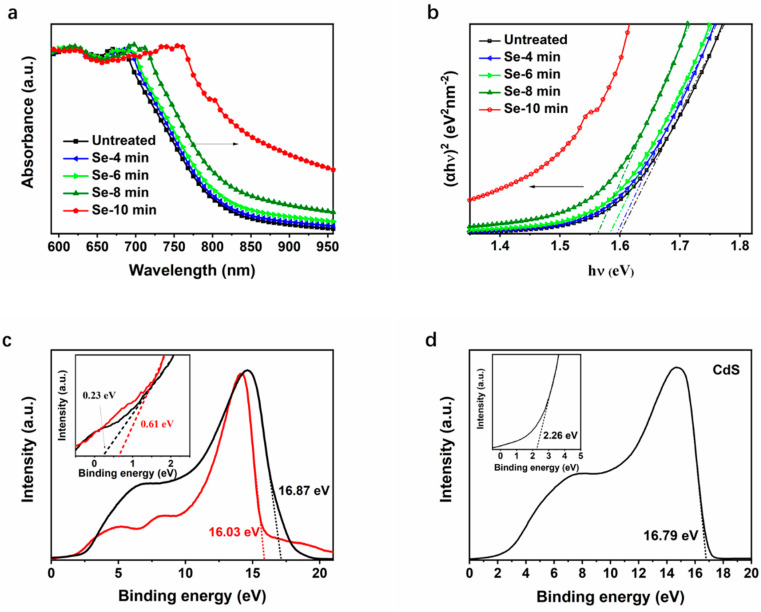
Optical properties and energy level analysis of CuSbS_2_ and CuSbS_2_ film: (**a**) UV–vis absorption spectra, (**b**) plots of (αhν)^2^ versus (hν); and UPS spectra of (**c**) CuSbS_2_(Se) selenized for 8 and 10 min and (**d**) CdS spectrum.

The effect of selenylation on the energy levels was subsequently investigated. Figure 6a shows the optical absorption spectra of absorbers heated in N_2_, and selenized for 4, 6, 8, and 10 min, respectively. Noteworthy, little difference is observed in absorption edges of the untreated film, film heated in N_2_, and films selenized for 4 min. The absorption spectra of films show a gradual red shift toward a longer wavelength with an increase in the selenylation duration. For the film selenized for 10 min, a much broader absorption is observed in the 800–950 nm range. Usually, the direct energy band gap (E_g_) of could be calculated by using the following formula [34]:

(4) $$\alpha =\left(A/h\nu \right)\times {\left(h\nu -E_{g}\right)}^{1/2},$$

with A is a constant, h is the Planck’s constant, and ν is the frequency of the incident photon. Figure 5 demonstrates that selenized CuSbS_2_ films belong to multiphase film, which could not be described by applying this formula in a strict sense. However, the Eg of CuSbS_2_(Se) could also be roughly estimated by using this formula by UV–vis absorption spectroscopy (Figure 7a) and model the linear zone of (αhν)^2^ versus (hν) when it was lightly selenized, as shown in Figure 7b. The untreated sample shows an E_g_ of 1.60 eV, which slightly increases to 1.58 eV as it heated in N_2_. The average E_g_ of CuSbS_2_(Se) decreased to 1.56 eV when it was selenized for 8 min. For the film selenized for 10 min, no linear zone could be fitted appropriately. However, undoubtedly, the average E_g_ is much lower than that for the film selenized for 10 min according to the variation trend. It indicates that, as a photovoltaic absorber, CuSbS_2_(Se) needs a longer selenylation time to create a suitable E_g_ to utilize more sunlight in the near-IR region. Currently, the problems such as improvement in the prevention of the appearance of pinholes or to find an effective passivation approach require urgent solutions.

UPS was used to further depict the energy level of CuSbS_2_(Se) [13,35]. The UPS spectra of CuSbS_2_ film heated in N_2_ and CuSbS_2_(Se) film selenized for 8 min are shown in Figure 7c. The UPS spectrum for CdS is shown in Figure 7d. The work function (W_F_), conduction band minimum (E_C_), and valence band maximum (E_V_) were determined based on the valence band onset (VBO) and cut-off region (E_cutoff_) of the UPS spectrum, and the results are listed in Table 4. Clearly, the VBO of CuSbS_2_ film heated in N_2_ is 0.27 eV, and it increases to 0.61 eV when selenized for 8 min. The W_F_ of CuSbS_2_ film heated in N_2_ is 4.34 eV, and it increases to 5.03 eV after selenylation. It can be concluded that the surface state and energy level of selenized CuSbS_2_ has been modified and would bring some effect on the carrier transport.

**Figure 8:**
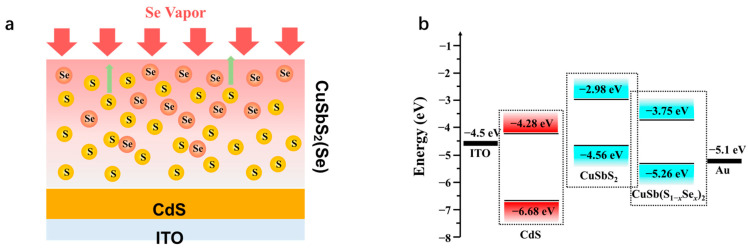
Energy band structure analysis of CdS/CuSbS_2_(Se): (**a**) a sketch of Se distribution in CuSbS_2_(Se) and (**b**) energy level of CdS/CuSbS_2_(Se) heterojunction interface.

As discussed earlier, the concentration of Se element gradient decreases from top to the deeper positions, and the corresponding distribution process is depicted in Figure 8a. For a lightly selenized CuSbS_2_(Se) absorber, the practical structure of device could be written as ITO/CdS/CuSbS_2_/CuSbS_2_(Se)/Au. Combining the results of UV–vis spectra and UPS analysis, the energy level of CdS/CuSbS_2_/CuSbS_2_(Se) p-i-n junction could be easily confirmed, as shown in Figure 8b. Notably, the E_C_ and E_V_ of CuSbS_2_(Se) are −3.75 eV and −5.26 eV, respectively, which are slightly lower than those of CuSbS_2_ and match better with the CdS buffer layer. The CuSbS_2_(Se)/CdS has a lower energy barrier for both holes and free electrons transport to anode and cathode, respectively. However, little Se could distribute to the interface of p–n junction in a short selenylation duration; therefore, CuSbS_2_ inevitably exists between CuSbS_2_(Se) and CdS. Both holes and free electrons would be blocked more or less by the CuSbS_2_ layer during their transport process. Thus, to further improve the performance of CdS/CuSbS_2_ solar cells, the CuSbS_2_ layer should be reduced to the maximum extent in order to avoid the destruction of the film structure.

## 4. Conclusions

In this study, CuSbS_2_(Se) films with a high Se substitution rate (~39%) were successfully prepared using porous CuSbS_2_ films deposited by spray pyrolysis followed by a post-treatment in Se vapor. The CuSbS_2_ device shows a significant improvement from 0.12% to 0.90%. Two major findings were concluded: First, selenylation leads to an obvious reduction of the carrier recombination center, which is related to deep-level V_S_ and antisite defects created by the loss of S during processing. Second, selenylation leads to the modification of the band structure and energy level of CuSbS_2_ films. The average E_g_ decreases from 1.58 to below 1.51 eV, and the E_C_ and E_V_ decrease from −2.98 and −4.56 eV to −3.75 and −5.26 eV, respectively, accompanied by a reduction of the energy barrier for both holes and free electrons transport to anode and cathode, respectively. Moreover, elemental analysis results reveal a gradient distribution for Se from the top surface to the deep region. Unselenized CuSbS_2_ layer existing in CuSbS_2_(Se) has some bad effect on the carrier transport. Thus, in order to further improve the performance of CdS/CuSbS_2_ solar cells, a more sufficient selenylation should be carried out and CuSbS_2_ layer should be reduced as much as possible. Undeniably, a lot more systematic explorations are still demanded to investigate an efficient selenylation technique that would not destroy the structure of the CuSbS_2_ films, which will be pursued in the future work.

## Figures and Tables

**Table 1:** The detailed photovoltaic parameters of CuSbS_2_ and CuSbS_2_ (Se) solar cells selenized for varied durations.

Sample	*V_OC_* (V)	*J_SC_* (mA cm^−2^)	FF	PCE (%)
Untreated	0.31 ± 0.02	1.41 ± 0.24	0.28 ± 0.03	0.12 ± 0.06
A-8 min	0.54 ± 0.01	2.60 ± 0.14	0.32 ± 0.02	0.46 ± 0.05
Se-4 min	0.35 ± 0.01	2.83 ± 0.32	0.30 ± 0.03	0.28 ± 0.07
Se-6 min	0.47 ± 0.01	3.70 ± 0.35	0.32 ± 0.03	0.57 ± 0.12
Se-8 min	0.48 ± 0.02	4.70 ± 0.41	0.39 ± 0.02	0.90 ± 0.14
Se-10 min	0.42 ± 0.04	2.00 ± 0.46	0.34 ± 0.04	0.31 ± 0.17

**Table 2:** Calculation parameters from the fitted impedance spectra.

Circuit Element	Sample					
	Untreated	N_2_-8 min	Se-4 min	Se-6 min	Se-8 min	Se-10 min
*R1* (Ω)	265.7	219.7	212.5	112.5	58.7	178.5
*Rce* (Ω)	6.17 × 10^5^	2.66 × 10^5^	1.87 × 10^5^	0.82 × 10^5^	0.66 × 10^5^	3.87 × 10^5^
*CPE1-T* (F cm^−2^)	0.23 × 10^−9^	1.32 × 10^−9^	1.61 × 10^−9^	6.81 × 10^−9^	9.12 × 10^−9^	0.78 × 10^−9^
*τ_avg_*_1_ (S)	1.42 × 10^−4^	3.51 × 10^−4^	3.01 × 10^−4^	5.58 × 10^−4^	6.01 × 10^−4^	3.01 × 10^−4^
*CPE1-P*	0.85	0.90	0.87	0.90	0.91	0.86
*Rrce* (Ω)	0.8 × 10^5^	1.2 × 10^5^	1.0 × 10^5^	5.5 × 10^5^	8.1 × 10^5^	4.3 × 10^5^
*CPE2-T* (F cm^−2^)	1.52 × 10^−5^	3.17 × 10^−5^	5.58 × 10^−5^	6.16 × 10^−5^	7.06 × 10^−5^	2.38 × 10^−5^
*τ_avg_*_2_ (S)	1.216	3.80	5.58	33.88	57.186	10.234
*CPE2-P*	0.93	0.93	0.94	0.94	0.96	0.89

**Table 3:** Global elemental analysis of Cu, Sb, S, and Se elements in CuSbS_2_(Se) samples selenized for different durations.

Sample	Composition (at.%)				Compositional Ratio (%)		
	Cu	Sb	S	Se	S/(S + Se)	Se/(S + Se)	(S + Se)/(Cu + Sb)
Untreated	25.69	25.54	48.77	0	1	0	0.95
Se-4 min	25.44	25.28	49.22	0.06	1	0	0.97
Se-6 min	25.43	25.17	46.33	2.77	0.94	0.06	0.97
Se-8 min	25.27	25.16	37.92	11.65	0.76	0.24	0.98
Se-10 min	25.57	25.30	29.92	19.21	0.61	0.39	0.97

**Table 4:** Parameters calculated from the UPS spectra.

Sample	E_g_ (eV)	VBO (eV)	W_F_ (eV)	E_C_ (eV)	E_V_ (eV)
CuSbS_2_	1.58	0.27	4.34	−2.98	−4.56
CuSbS_2_(Se)	1.51	0.61	5.03	−3.75	−5.26
CdS	2.4	2.26	4.42	−4.28	−6.68

## Data Availability

The data presented in this study are available on a reasonable request from the corresponding author.
